# Supplementary material for: Meta-learning biologically plausible plasticity rules with random feedback pathways
Source: Nat Commun. 2023 Mar 31;14:1805. doi: 10.1038/s41467-023-37562-1 (PMC10066328; doi:10.1038/s41467-023-37562-1)
Supplement: Supplementary file 1 — Supplementary Information [file 41467_2023_37562_MOESM1_ESM.pdf]

Supplementary Information

Meta-Learning Biologically Plausible Plasticity Rules with Random Feedback Pathways

Navid Shervani-Tabar[*,1] and Robert Rosenbaum[1]

[1]*Department of Applied and Computational Mathematics and Statistics, University of Notre Dame, Notre Dame, IN 46556, USA*
[*]*Corresponding author: nshervan@nd.edu*

1 Performance of DFA

Figure 1 illustrates that the Feedback Alignment model [1] is less effective than the backprop model when training deep networks with a continuous data stream. To be more precise, the backprop model begins learning immediately at the start of training, while the Feedback Alignment model takes around 2000 training data points before it starts to learn. Additionally, the rate of learning for the Feedback Alignment model is slower.

In an attempt to improve the Feedback Alignment model's performance, the Direct Feedback Alignment (DFA) method [2] proposed altering the backward connections to directly transmit errors from the output layer $\mathbf{y}_L$ to the upstream layers $\mathbf{y}_\ell$. The modulating signals in this modified model are calculated as

$$\mathbf{e}_\ell = \boldsymbol{B}_{L,\ell}\mathbf{e}_L \odot \sigma'(\mathbf{z}_\ell), \tag{S.1}$$

with

$$\mathbf{e}_L = \frac{\partial \mathcal{L}}{\partial \mathbf{z}_L}. \tag{S.2}$$

In this formulation, $\boldsymbol{B}_{L,\ell} \in \mathbb{R}^{\dim(\mathbf{y}_\ell)\times\dim(\mathbf{y}_L)}$, where $\dim(\mathbf{y}_\ell)$ represents the dimensionality of the activation $\mathbf{y}_\ell$.

As shown in Fig. S1, incorporating direct feedback connections to the Feedback Alignment method speeds up learning, and the model's accuracy improves after 1000 training data points. However, even with this modification, the network's performance is still lower than that of the backprop model. Figure S1 further compares the DFA model with the Feedback Alignment model trained with the $\mathcal{F}^{\text{bio}}$ plasticity rule (Eq. 7) and shows that the improved plasticity rule outperforms the DFA model.

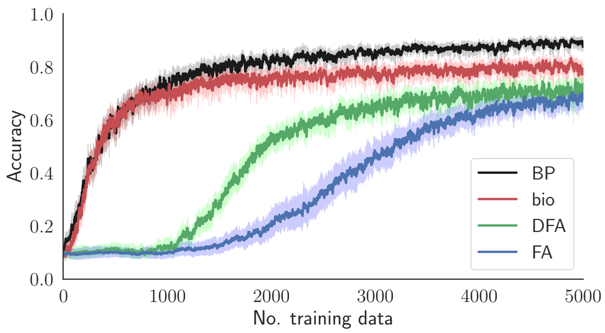

Figure S1: **Performance of benchmark learning schemes** while training a 5−layer fully-connected classifier network on MNIST digits [3] for a 10-way classification task. The plot demonstrates accuracy versus the number of training data for Feedback Alignment (FA) [1], Direct Feedback Alignment (DFA) [2], and backprop (BP) [4] methods, compared to the discovered biologically plausible plasticity rule $\mathcal{F}^{\text{bio}}$ (bio; Eq. 7).

2 Performance of the $\mathcal{F}^{\text{bio}}$

Fig. S2 demonstrates the classifier's performance with $\mathcal{F}^{\text{bio}}$ within 600 iterations of the meta-optimizer. Comparing the loss and accuracy of $\mathcal{F}^{\text{bio}}$ with $\mathcal{F}^{0}$ via feedback alignment in Fig. S2a and Fig. S2b, respectively, shows a significant boost in learning through $\mathcal{F}^{\text{bio}}$. Figure S2c further shows improvement in the alignment of the modulating signals with those of the backprop. These angles are reduced the most in the deeper layers. Lastly, Fig. S2d illustrates the progress of the meta-parameters. We observe that the plasticity coefficients converge in about 200 episodes.

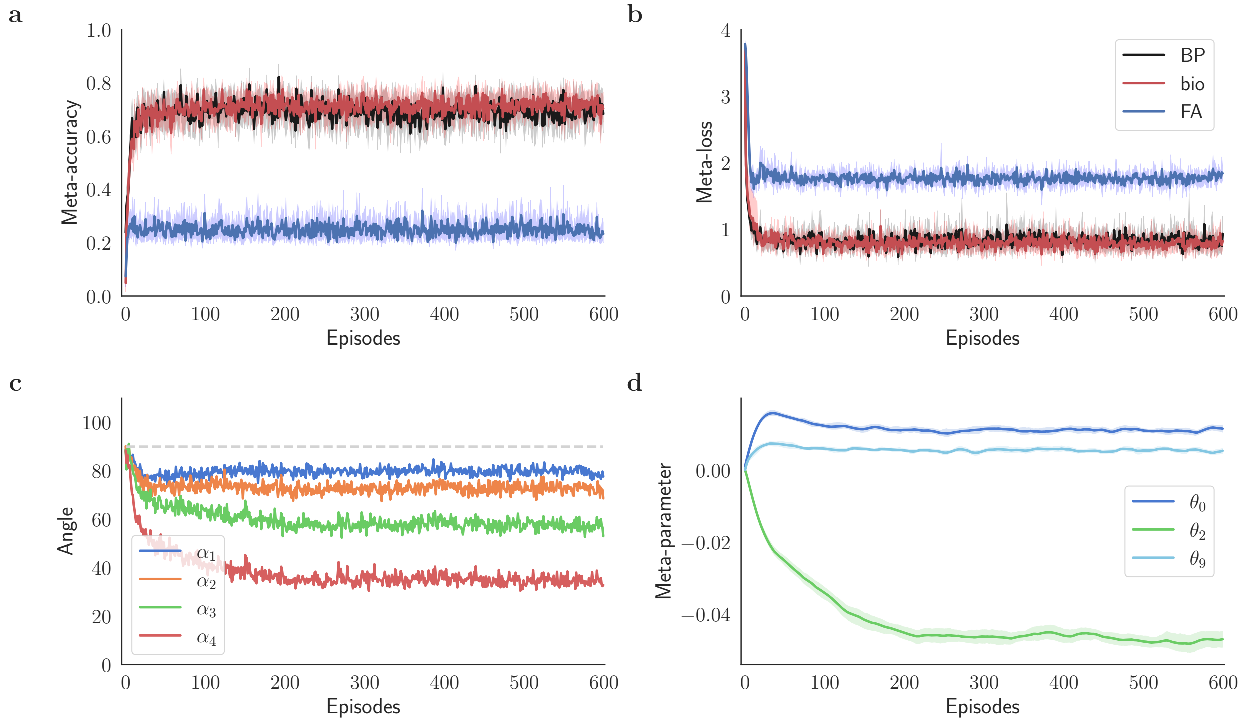

Figure S2: **Performance of the classifier network trained with $\mathcal{F}^{\text{bio}}$ plasticity rule:** Comparison between (**a**) meta-accuracy and (**b**) meta-loss of $\mathcal{F}^{\text{bio}}$ rule (bio; Eq. 7) with $\mathcal{F}^{0}$ via feedback alignment (FA) and backprop (BP), (**c**) alignment angles α_ℓ between modulating signals of $\mathcal{F}^{\text{bio}}$ and backprop, and (**d**) convergence of the plasticity meta-parameters $\boldsymbol{\Theta} = \{\theta_0, \theta_2, \theta_9\}$. While the term $\mathcal{F}^{\text{bio}}$ was discovered by regularizing the meta-loss with the penalty term in Eq. 6 (See Methods), λ is set to zero for the illustrations in this figure for the uncovered rule.

3 Data flow in $\mathcal{F}^{\text{eHebb}}$

Table 1 demonstrates the effect of the Hebbian-like error plasticity term (Eq. 8) on the alignment angles of the modulator signals. Here, we explain these improvements by illustrating $\mathcal{F}^{\text{eHebb}}$'s influence on the feedback pathway's interactions with the forward path. To set the baseline, Fig. S3a employs the plasticity rule

$$\mathcal{F}^{0}(\boldsymbol{\Theta}) = -\theta_0 \mathbf{e}_\ell \mathbf{y}_{\ell-1}^T \tag{S.3}$$

to train the network (row 1 in Tab. 1), where $\mathbf{e}_\ell$ is transmitted through random feedback pathways. First, information from backward connections $\boldsymbol{B}_{2,1}$ and $\boldsymbol{B}_{3,2}$ (through $\boldsymbol{B}_{2,1}$) flows into $\boldsymbol{W}_{0,1}$ via Eqs. 3 and S.3. Similarly, information from $\boldsymbol{B}_{3,2}$ flows into $\boldsymbol{W}_{1,2}$ during the weight update. Then in the forward pass, information from $\boldsymbol{W}_{0,1}$ and $\boldsymbol{W}_{1,2}$ are propagated forward into $\boldsymbol{W}_{2,3}$. Table 1 shows that this flow of information does not sufficiently adjust $\boldsymbol{W}$ for a good alignment of the teaching signals, particularly in online training with limited data.

In Fig. S3b, we add the Hebbian-style error term to update $\boldsymbol{W}_{2,3}$ using $\mathcal{F}^{\text{eHebb}}$, while the rest of the network is trained with $\mathcal{F}^{0}$ through feedback alignment (Tab. 1, row 3). The information flow to $\boldsymbol{W}_{0,1}$ and $\boldsymbol{W}_{1,2}$ stays the same; however, $\mathcal{F}^{\text{eHebb}}$ introduces an auxiliary information channel from $\boldsymbol{B}_{3,2}$ to $\boldsymbol{W}_{2,3}$. As presented in Tab. 1, this supplementary channel results in a better alignment of $\mathbf{e}_2$ with the corresponding error vector transmitted via backprop.

Figure S3c repeats this experiment with $\boldsymbol{W}_{1,2}$ updated using $\mathcal{F}^{\text{eHebb}}$ while other layers are updated with $\mathcal{F}^0$ with feedback alignment (Tab. 1, row 2). Although $\boldsymbol{W}_{0,1}$ is updated with the same flow of information as Fig. S3b, there is a new flow from $\boldsymbol{B}_{2,1}$ to $\boldsymbol{W}_{1,2}$, which improves $\mathbf{e}_1$'s alignment. Note that better alignment of $\mathbf{e}_1$ results in more backprop-like weight update, which subsequently improves data propagation to the downstream layers. As a result, the alignments in the downstream layers are slightly improved as well, even with the vanilla $\mathcal{F}^0$ plasticity rule with feedback alignment updating them. This behavior is similar to the reduced alignment angles in Fig. 7c, where $\mathcal{F}^{\text{Oja}}$ positively affects the alignments by improving the forward data propagation.

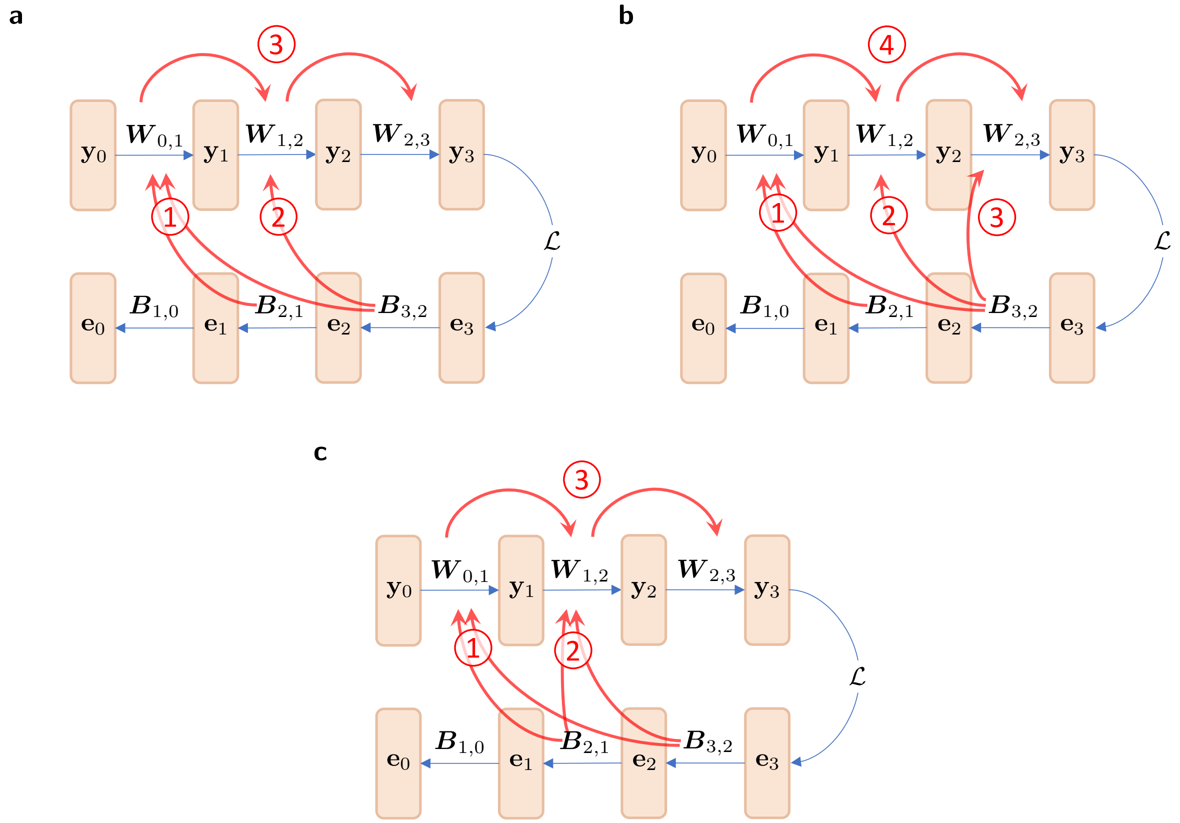

Figure S3: **Interactions between feedback and forward pathways using $\mathcal{F}^{\text{eHebb}}$:** (**a**) All layers trained with the rule $\mathcal{F}(\boldsymbol{\Theta}) = \theta_0\mathcal{F}^0$ via feedback alignment. Information from $\boldsymbol{B}_{3,2}$ and $\boldsymbol{B}_{2,1}$ is transmitted to $\boldsymbol{W}_{0,1}$ through the $\mathcal{F}^0$ plasticity rule (①), which then passes on to $\boldsymbol{W}_{1,2}$ and $\boldsymbol{W}_{2,3}$ (③). Meanwhile, information from $\boldsymbol{B}_{3,2}$ is transmitted to $\boldsymbol{W}_{1,2}$ (②), which is then propagated to $\boldsymbol{W}_{2,3}$ after the forward propagation. (**b**) $\boldsymbol{W}_{2,3}$ is updated using $\mathcal{F}^{\text{eHebb}}(\boldsymbol{\Theta}) = \theta_0\mathcal{F}^0 + \theta_2\mathcal{F}^2$, while $\boldsymbol{W}_{0,1}$ and $\boldsymbol{W}_{1,2}$ are trained with the rule $\mathcal{F}(\boldsymbol{\Theta}) = \theta_0\mathcal{F}^0$ via feedback alignment. Plasticity rule $\mathcal{F}^0$ transmits information from $\boldsymbol{B}_{2,1}$ and $\boldsymbol{B}_{3,2}$ to $\boldsymbol{W}_{0,1}$ (①) and from $\boldsymbol{B}_{3,2}$ to $\boldsymbol{W}_{1,2}$ (②). This information is propagated to their downstream layers after the forward path (④). Concurrently, an additional channel established by $\mathcal{F}^2$ explicitly propagates the information from $\boldsymbol{B}_{3,2}$ to $\boldsymbol{W}_{2,3}$ (③). (**c**) $\boldsymbol{W}_{0,1}$ and $\boldsymbol{W}_{2,3}$ use the plasticity rule $\mathcal{F}(\boldsymbol{\Theta}) = \theta_0\mathcal{F}^0$ via feedback alignment, and $\boldsymbol{W}_{1,2}$ utilizes $\mathcal{F}^{\text{eHebb}}(\boldsymbol{\Theta}) = \theta_0\mathcal{F}^0 + \theta_2\mathcal{F}^2$. $\mathcal{F}^0$ communicates information from $\boldsymbol{B}_{2,1}$ and $\boldsymbol{B}_{3,2}$ to $\boldsymbol{W}_{0,1}$ (①), which then is propagated to the downstream layers (③). Meanwhile, the $\mathcal{F}^0$ rule in $\mathcal{F}^{\text{eHebb}}$ disseminates information from $\boldsymbol{B}_{3,2}$ to $\boldsymbol{W}_{1,2}$, while $\mathcal{F}^2$ in $\mathcal{F}^{\text{eHebb}}$ establishes a direct route to transmit information from $\boldsymbol{B}_{2,1}$ to $\boldsymbol{W}_{1,2}$ (②). The ensuing forward propagation from $\boldsymbol{W}_{1,2}$ to the downstream layers continues as usual. In all graphs, blue arrows represent the propagation of data through the forward or backward path, while the red arrow represents the flow of information from the backward pathway to the forward connections.

4 Expectation of Hebbian-style error-based plasticity

Assume that the entries of $\boldsymbol{B}_{\ell+1,\ell}$ are i.i.d. with expectation zero and independent from the entries of $\mathbf{e}_{\ell+1}$. Also assume that the entries of $\mathbf{e}_\ell$ have variance σ_ℓ^2. In this Supplementary section, we show that

$$\mathbb{E}\left[\mathbf{e}_\ell \mathbf{e}_{\ell-1}^T \,\middle|\, \boldsymbol{B}_{\ell,\ell-1}\right] = \sigma_\ell^2 \boldsymbol{B}_{\ell,\ell-1}^T \tag{S.4}$$

We must first show that $\mathbb{E}[(\mathbf{e}_\ell)_j)] = 0$ and $\mathbb{E}[(\mathbf{e}_\ell)_i(\mathbf{e}_\ell)_j)] = 0$ when $i \neq j$ by computing

$$\begin{aligned}
\mathbb{E}\left[(\mathbf{e}_\ell)_j\right] &= \mathbb{E}\left[\left(\sum_k (\boldsymbol{B}_{\ell+1,\ell})_{j,k}\,(\mathbf{e}_{\ell+1})_k\right)\right] && \text{(S.5)}\\
&= \sum_k \mathbb{E}\left[(\boldsymbol{B}_{\ell+1,\ell})_{j,k}\right]\mathbb{E}\left[(\mathbf{e}_{\ell+1})_k\right] && \text{(S.6)}\\
&= 0. && \text{(S.7)}
\end{aligned}$$

Now, assume that $i \neq j$ and compute

$$\begin{aligned}
\mathbb{E}\left[(\mathbf{e}_\ell)_i(\mathbf{e}_\ell)_j\right] &= \mathbb{E}\left[\left(\sum_k (\boldsymbol{B}_{\ell+1,\ell})_{i,k}(\mathbf{e}_{\ell+1})_k\right)\left(\sum_{k'} (\boldsymbol{B}_{\ell+1,\ell})_{j,k'}(\mathbf{e}_{\ell+1})_{k'}\right)\right] && \text{(S.8)}\\
&= \sum_{k,k'}\left(\mathbb{E}\big[\,(\boldsymbol{B}_{\ell+1,\ell})_{i,k}\,(\boldsymbol{B}_{\ell+1,\ell})_{j,k'}\,\big]\mathbb{E}\big[\,(\mathbf{e}_{\ell+1})_k\,(\mathbf{e}_{\ell+1})_{k'}\,\big]\right) && \text{(S.9)}\\
&= 0 && \text{(S.10)}
\end{aligned}$$

where the last line follows from the assumptions that $i \neq j$ and $\boldsymbol{B}_{\ell+1,\ell}$ has independent entries.

Now we can derive Eq. (S.4) as follows

$$\begin{aligned}
\mathbb{E}\left[\left(\mathbf{e}_\ell\mathbf{e}_{\ell-1}^T\right)_{i,k}\,\middle|\,\boldsymbol{B}_{\ell,\ell-1}\right] &= \mathbb{E}\left[\left(\mathbf{e}_\ell\mathbf{e}_\ell^T\boldsymbol{B}_{\ell,\ell-1}^T\right)_{i,k}\,\middle|\,\boldsymbol{B}_{\ell,\ell-1}\right] && \text{(S.11)}\\
&= \mathbb{E}\left[\sum_{j=1}^n \left(\mathbf{e}_\ell\mathbf{e}_\ell^T\right)_{i,j}\left(\boldsymbol{B}_{\ell,\ell-1}^T\right)_{j,k}\,\middle|\,\boldsymbol{B}_{\ell,\ell-1}\right] && \text{(S.12)}\\
&= \sum_{j=1}^n \mathbb{E}\left[(\mathbf{e}_\ell)_i\,(\mathbf{e}_\ell)_j\left(\boldsymbol{B}_{\ell,\ell-1}^T\right)_{j,k}\,\middle|\,\boldsymbol{B}_{\ell,\ell-1}\right] && \text{(S.13)}\\
&= \mathbb{E}\left[(\mathbf{e}_\ell)_i\,(\mathbf{e}_\ell)_i\left(\boldsymbol{B}_{\ell,\ell-1}^T\right)_{i,k}\,\middle|\,\boldsymbol{B}_{\ell,\ell-1}\right] && \text{(S.14)}\\
&= \sigma_\ell^2\left(\boldsymbol{B}_{\ell,\ell-1}^T\right)_{i,k} && \text{(S.15)}
\end{aligned}$$

The last two lines follow from the fact that whenever $i \neq j$, the expectation is equal to zero. Eq. (S.4) follows directly.

5 Performance of the $\mathcal{F}^{\mathbf{Oja}}$ on FashionMNIST

In the main article, we examine how Oja's rule improves learning in the Feedback Alignment model (Fig. 7). In this section, we demonstrate the effectiveness of Oja's rule on a different dataset by using the FashionMNIST [5] to train a classifier model. Figure S4 illustrates that

introducing the Oja's rule (Eq. 10) substantially enhances learning across different datasets when the model is trained with random feedback connections.

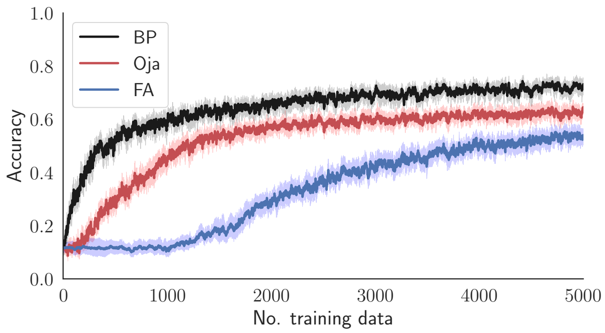

Figure S4: **Performance of benchmark learning schemes** while training a 5−layer fully-connected classifier network on FashionMNIST dataset [5] for a 10-way classification task. The plot demonstrates accuracy versus the number of training data for Feedback Alignment (FA) [1] and backprop (BP) [4] methods, compared to $\mathcal{F}^{\mathrm{Oja}}$ (Oja; Eq. 10).

6 Performance of alternative penalization methods

In Results, we proposed using L1 regularization on the meta-loss to decrease redundancy within the update rules. As shown in Fig. 4d, this technique leads to a sparser set of meta-parameters and acts as a model selection method, identifying the most effective plasticity rules.

In Fig. S5, we examine the impact of alternative regularization methods on the meta-learning algorithm by comparing the performance of models with no regularization and L2 regularization. When using no regularization in the meta-learning, the algorithm eliminates update terms negatively impacting the learning. However, another set of plasticity rules may individually improve the results, but when these rules are considered in a set, other terms may be more beneficial for the optimization process. Nevertheless, the model still includes them in the final meta-optimized learning rule. As seen in Fig. S5a, the model has identified seven plasticity terms, making it impractical to investigate each of these terms individually.

As an alternative, Fig. S5b shows the results of using L2 regularization

$$\mathcal{L}_{\mathrm{meta}}(\boldsymbol{\Theta}) = \mathcal{L}(f_{\boldsymbol{W}}(\boldsymbol{X}_{\mathrm{query}}), \boldsymbol{Y}_{\mathrm{query}}) + \lambda \|\boldsymbol{\Theta}\|_2. \tag{S.16}$$

Unlike L1 regularization, L2 tends to decrease all parameters but does not return sparse solutions and is unsuitable for feature selection. In other words, even though L2 regularization reduces the values of all parameters, it does not eliminate the redundant or less influential plasticity terms with large meta-parameters from the final solution. Table S1 summarizes the resulting meta-parameters using each meta-loss regularization approach.

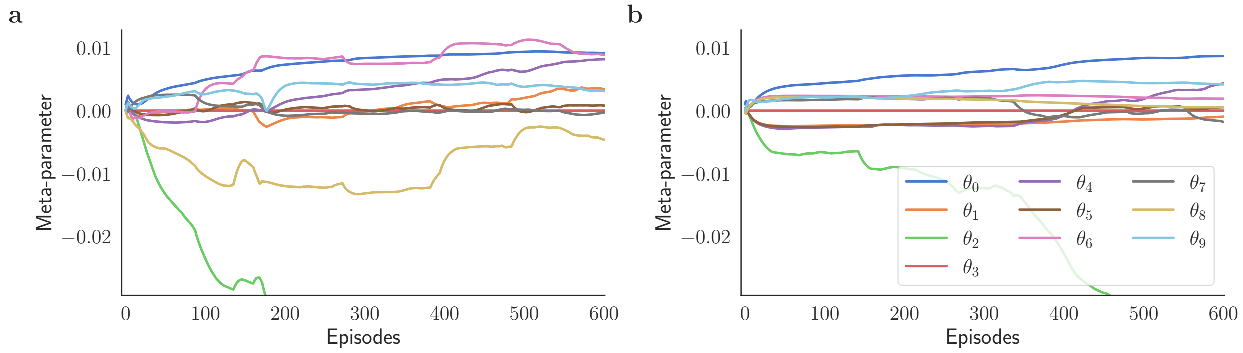

Figure S5: **L1 improves feature selection in the meta-learning model:** Performance of different penalization methods while training a 5−layer fully-connected classifier network on EMNIST digits [6]. Evolution of meta-parameters $\mathbf{\Theta} = \{\theta_r\}_{0\leq r\leq 9}$ for the pool of learning rules defined in Methods using (**a**) no penalization, (**b**) L2 penalized meta-loss (Eq. S.16).

Meta-parameter	No regularization	L1	L2
θ_0	0.915×10^{-2}	0.978×10^{-2}	0.867×10^{-2}
θ_1	0.343×10^{-2}	0.075×10^{-2}	-0.095×10^{-2}
θ_2	-3.654×10^{-2}	-2.361×10^{-2}	-3.305×10^{-2}
θ_3	0.000×10^{-2}	0.000×10^{-2}	0.000×10^{-2}
θ_4	0.816×10^{-2}	0.087×10^{-2}	0.430×10^{-2}
θ_5	0.081×10^{-2}	0.026×10^{-2}	0.054×10^{-2}
θ_6	0.887×10^{-2}	0.019×10^{-2}	0.188×10^{-2}
θ_7	-0.031×10^{-2}	0.031×10^{-2}	-0.177×10^{-2}
θ_8	-0.459×10^{-2}	-0.064×10^{-2}	0.053×10^{-2}
θ_9	0.313×10^{-2}	0.415×10^{-2}	0.417×10^{-2}

Table S1: **Effect of regularization methods on feature selection in meta-learning the plasticity rule:** The resulting values of meta-parameters $\mathbf{\Theta} = \{\theta_r\}_{0\leq r\leq 9}$ for the pool of learning rules defined in Methods are displayed using no penalization (Eq. 6 with $\lambda = 0$; Fig. S5a), L1 (Eq. 6; Fig. 4d), and L2 penalized meta-loss (Eq. S.16; Fig. S5b). The values reported for L1 are averaged across 20 trials. All values are reported for the final episode $\mathcal{E} = 600$.

7 Performance of alternative backward initialization

As mentioned in Methods, the Xavier initialization method was used to randomly sample forward and backward connections from a uniform distribution

$$\boldsymbol{B}_{\ell+1,\ell}, \boldsymbol{W}_{\ell,\ell+1} \sim \mathcal{U}\left(-\sqrt{\frac{6}{\dim(\mathbf{y}_\ell)+\dim(\mathbf{y}_{\ell+1})}}, \sqrt{\frac{6}{\dim(\mathbf{y}_\ell)+\dim(\mathbf{y}_{\ell+1})}}\right) \tag{S.17}$$

throughout the study, where $\dim(\mathbf{y}_\ell)$ is the dimension of the activation $\mathbf{y}_\ell$. Nevertheless, the findings presented in this work do not depend on the initialization method of the backward connections.

To illustrate this, we conducted an experiment where we employed the normal Xavier initialization method

$$\boldsymbol{B}_{\ell+1,\ell} \sim \mathcal{N}\left(0, \frac{2}{\dim(\mathbf{y}_\ell) + \dim(\mathbf{y}_{\ell+1})}\right) \tag{S.18}$$

to sample initial values for the backward connections. The forward connections were initialized using a uniform distribution as before (Eq. S.17). Figure S6 shows that the proposed $\mathcal{F}^{\text{bio}}$ plasticity rule can successfully train the model using different methods for initializing the backward connections.

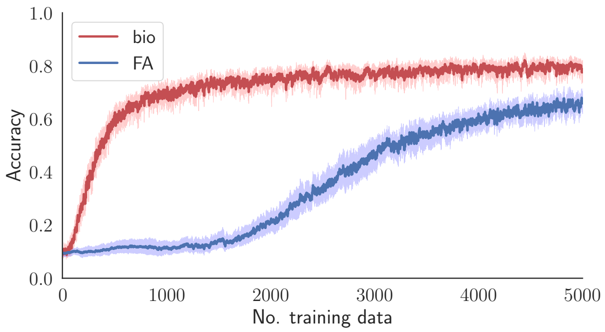

Figure S6: $\mathcal{F}^{\text{bio}}$ **trains effectively under different initialization of the feedback:** Accuracy of a 5-layer classifier network trained on MNIST dataset [3] to perform a 10-way classification task using Feedback Alignment (FA) [1] compared to the proposed $\mathcal{F}^{\text{bio}}$ plasticity rule (bio) outlined in Eq. 7. The backward connections were initialized in both tests using the normal Xavier initialization method (Eq. S.18).

8 Inter-treatment variation

Throughout the paper, we examine the variations within each plasticity rule by calculating the confidence intervals. To determine if the improvements in accuracy are statistically significant, we use the Mann-Whitney U test to compare two sets of data: the accuracy of trials using the FA method and the modified plasticity rule. Samples are taken at the end of each episode and represent the accuracy of the model trained with different initial weights and feedback connection values. We chose the Mann-Whitney U test over the t-test as it does not assume a Gaussian distribution within the groups.

We begin by hypothesizing that the FA method trial samples show lower accuracy than that of the modified plasticity rule. We utilize 20 samples from each group. The results, illustrated in Fig. S7, indicate that the p-value falls below 5% within fewer than 100 episodes in every example. Our findings indicate strong evidence against the null hypothesis, providing statistical support for the performance gain using the proposed plasticity rules.

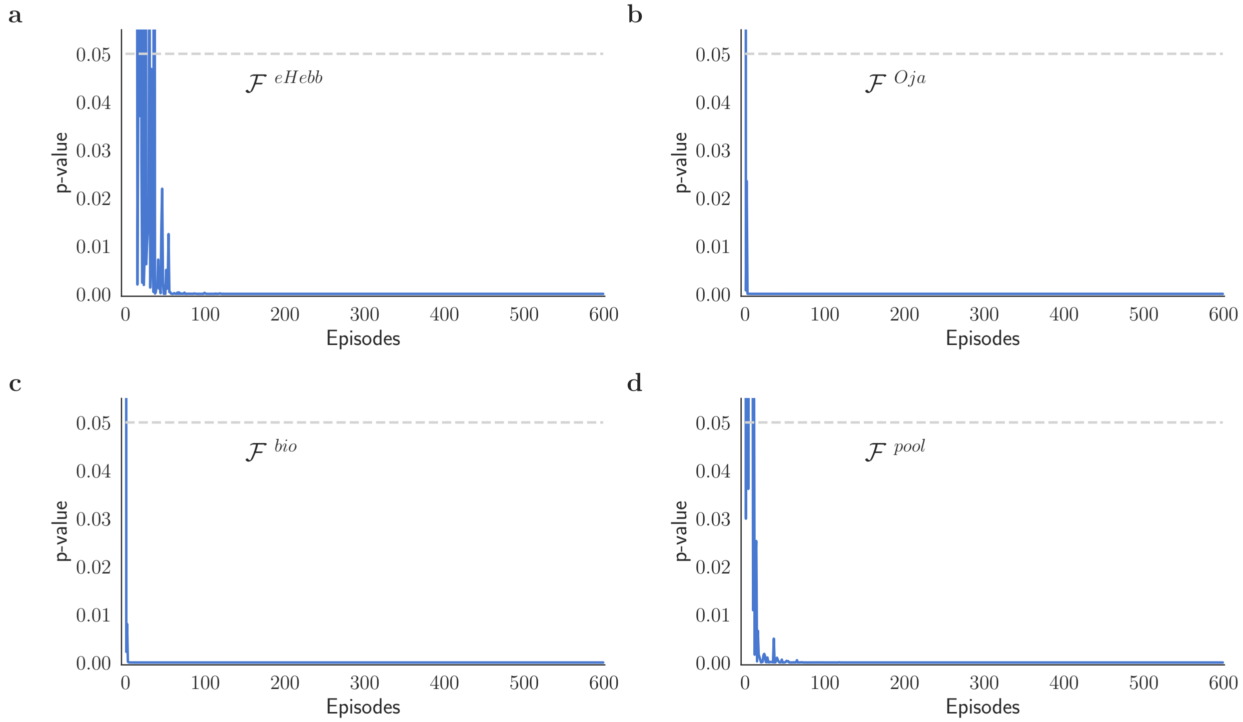

Figure S7: **The performance gain obtained with the modified plasticity rules is statistically significant:** The p-value of the one-sided Mann-Whitney test over 600 meta-optimization episodes, comparing samples from trials using the FA method to those using (**a**) $\mathcal{F}^{\mathrm{eHebb}}$, (**b**) $\mathcal{F}^{\mathrm{Oja}}$, (**c**) $\mathcal{F}^{\mathrm{bio}}$, and (**d**) $\mathcal{F}^{\mathrm{pool}}$ plasticity rules.

Supplementary References

[1] T. P. Lillicrap, D. Cownden, D. B. Tweed, and C. J. Akerman. Random synaptic feedback weights support error backpropagation for deep learning. *Nature communications*, 7(1):1–10, 2016.

[2] A. Nøkland. Direct feedback alignment provides learning in deep neural networks. *Advances in neural information processing systems*, 29, 2016.

[3] Y. LeCun, L. Bottou, Y. Bengio, and P. Haffner. Gradient-based learning applied to document recognition. *Proceedings of the IEEE*, 86(11):2278–2324, 1998.

[4] D. E. Rumelhart, G. E. Hinton, and R. J. Williams. Learning representations by back-propagating errors. *nature*, 323(6088):533–536, 1986.

[5] H. Xiao, K. Rasul, and R. Vollgraf. Fashion-mnist: a novel image dataset for benchmarking machine learning algorithms. *arXiv preprint arXiv:1708.07747*, 2017.

[6] G. Cohen, S. Afshar, J. Tapson, and A. Van Schaik. Emnist: Extending mnist to handwritten letters. In *2017 international joint conference on neural networks (IJCNN)*, pages 2921–2926. IEEE, 2017.
